# Correction: CuI-mediated synthesis of 1-aryl-5,6,7-trimethoxybenzimidazoles as potent antitubulin agents

**DOI:** 10.1039/d3ra90046k

**Published:** 2023-05-16

**Authors:** Cong-Min Peng, Shih-Wei Wang, Yi-Lin Hwang, Wen-Chun Sun, Li-Pin Chiu, Yi-Ting Liu, Yu-Wei Lai, Hsueh-Yun Lee

**Affiliations:** a School of Pharmacy, College of Pharmacy, Taipei Medical University Taipei Taiwan hyl@tmu.edu.tw; b Institute of Biomedical Sciences, MacKay Medical College New Taipei City Taiwan; c Department of Medicine, MacKay Medical College New Taipei City Taiwan; d School of Pharmacy, College of Pharmacy, Kaohsiung Medical University Kaohsiung Taiwan; e Division of Colon and Rectal Surgery, Department of Surgery, MacKay Memorial Hospital Taipei Taiwan; f Division of General Surgery, Taipei City Hospital Chushing Branch Taipei Taiwan; g General Education Center, University of Taipei Taipei Taiwan; h Division of Urology, Taipei City Hospital Renai Branch Taipei Taiwan dai77@tpech.gov.tw; i Department of Urology, School of Medicine and Shu-Tien Urological Science Research Center, National Yang Ming Chiao Tung University Taipei Taiwan; j PhD Program in Drug Discovery and Development Industry, College of Pharmacy, Taipei Medical University Taipei Taiwan; k TMU Research Center of Cancer Translational Medicine, Taipei Medical University Taipei Taiwan

## Abstract

Correction for ‘CuI-mediated synthesis of 1-aryl-5,6,7-trimethoxybenzimidazoles as potent antitubulin agents’ by Cong-Min Peng *et al.*, *RSC Adv.*, 2023, **13**, 13169–13176, https://doi.org/10.1039/D3RA01927F.

The authors regret that the one of the affiliations (affiliation *h*) was incorrectly shown in the original manuscript. The corrected list of affiliations is as shown here.

The Royal Society of Chemistry apologises for these errors and any consequent inconvenience to authors and readers.

## Supplementary Material

